# Gynecologic and reproductive outcomes in fibrous dysplasia/McCune-Albright syndrome

**DOI:** 10.1186/s13023-019-1057-x

**Published:** 2019-04-29

**Authors:** Alison M. Boyce, Rachel K. Casey, Diana Ovejero Crespo, Cynthia M. Murdock, Andrea Estrada, Lori C. Guthrie, Beth A. Brillante, Veronica Gomez-Lobo, Lynette K. Nieman, Michael T. Collins

**Affiliations:** 1 Skeletal Disorders & Mineral Homeostasis Section, National Institute of Dental and Craniofacial Research, National Institutes of Health, 30 Convent Drive Room 218 MSC 4320, Bethesda, MD USA; 2 Division of Endocrinology & Diabetes, Children’s National Health System, 111 Michigan Ave NW, Washington, D.C, USA; 3 Division of Pediatric and Adolescent Gynecology, Department of Obstetrics and Gynecology, MedStar Washington Hospital Center, Children’s National Health System, Washington, D.C., USA; 4 Pediatric and Adolescent Gynecology, Inova Health System, Pediatric Specialists of Virginia, Fairfax, VA USA; 5 Musculoskeletal Research Unit, Hospital del Mar Institute of Medical Investigation (IMIM), Barcelona, Spain; 6 National Research Council, Institute of Clinical Physiology, Lecce, Italy; 7 Program in Reproductive and Adult Endocrinology, The Eunice Kennedy Shriver National Institute of Child Health and Human Development, National Institutes of Health, Bethesda, MD USA; 8 Reproductive Medicine Associates of Connecticut, Norwalk, CT USA

**Keywords:** Ovarian cyst, Abnormal uterine bleeding, Gynecology, Fertility, Estrogen

## Abstract

**Background:**

Autonomous ovarian activation with recurrent estrogen-producing cysts is a hallmark feature of the rare bone and endocrine disorder fibrous dysplasia/McCune-Albright syndrome. Precocious puberty in girls with McCune-Albright syndrome has been well-described, however long-term effects on gynecologic and reproductive function are unknown. Concerningly, case reports have described poor skeletal outcomes associated with pregnancy in women with fibrous dysplasia.

**Methods:**

Thirty-nine women with fibrous dysplasia/McCune-Albright syndrome were evaluated as part of a natural history study. Clinical, radiographic, and biochemical data were reviewed. Women were contacted to obtain detailed menstrual and reproductive histories.

**Results:**

Abnormal uterine bleeding affected 77% of women (30/39), and was associated with severe anemia requiring blood transfusion in 3 cases. Nine women underwent hysterectomy for management of bleeding, including 67% (6/9) at the unusually young age of less than age 35 years. Infertility affected 43% of women (9/21), including 2 women who developed primary ovarian insufficiency after undergoing surgical treatment of ovarian cysts. Of 25 spontaneous pregnancies in 14 women, 35% (8) were unplanned. Among the 14 pregnancies, pregnancy was associated with no change in bone pain in 7 subjects (53%), increased bone pain in 4 subjects (31%), and decreased bone pain in 2 subjects (15%). No additional skeletal complications were reported during pregnancies.

**Conclusions:**

Women with fibrous dysplasia/McCune-Albright syndrome report a high prevalence of gynecologic morbidity and reduced fertility. There is no clear association between pregnancy and poor skeletal outcomes in this population.

## Introduction

McCune-Albright syndrome (MAS)(ORPHA:562) is a rare disorder arising from somatic gain-of-function mutations in Gα_s_ [1]. Disease presents along a broad spectrum that includes a variable combination of fibrous dysplasia of bone (FD), hyperpigmented skin macules, and hyperfunctioning endocrinopathies [2]. A hallmark feature of MAS is autonomous estrogen-secreting ovarian cysts [3]. Girls typically present in early childhood with signs of episodic estrogen exposure, including breast development, growth acceleration, and vaginal bleeding, which resolve in the interval between cysts [4, 5]. Gonadotropin levels are typically suppressed when estradiol levels are elevated; however, prolonged exposure to high estradiol levels can mature the hypothalamic pituitary axis, leading to secondary gonadotropin-dependent precocious puberty. Treatment with aromatase inhibitors, alone or in combination with gonadotropin releasing hormone agonists, is typically effective at preventing progressive pubertal development during childhood.

While the presentation, natural history, and clinical management of MAS-associated precocious puberty has been well-characterized, ovarian function in adulthood is poorly understood. It is unknown if women with a history of MAS-associated precocious puberty are at risk for gynecologic disease. Effects of persistent ovarian activation on fertility and childbearing, as well as the effects of pregnancy on skeletal outcomes, have not been determined. The obstetrical literature in FD/MAS is limited to case reports of skeletal complications during pregnancy, including increased FD-related bone pain [6, 7], aneurysmal bone cysts [6, 8, 9], and malignant transformation of FD lesions [10]. This has led some to speculate that pregnancy increases FD activity, placing women at risk for poor skeletal outcomes [6, 7, 11]. These knowledge gaps in gynecologic and reproductive outcomes are a significant source of concern for patients and families affected by FD/MAS.

The purpose of this study was to evaluate gynecologic and reproductive outcomes in a cohort of adult women with FD/MAS.

## Methods

Subjects were evaluated at the NIH Clinical Center between 1998 and 2015 as part of a longstanding FD/MAS natural history study. The protocol was approved by the NIDCR Institutional Review Board, and informed consent/assent was obtained from all subjects and/or their guardians. Subjects underwent history and physical exam, pelvic ultrasonography, and biochemistries to include LH, FSH, and estradiol. The diagnosis of FD/MAS was made on clinical grounds based on the presence of 2 or more characteristic features, with molecular testing as needed, according to previously published guidelines [2].

Attempts were made to contact all 90 women who had been seen in the natural history protocol to obtain detailed gynecologic and reproductive histories, and responses were received from 39 subjects. Five interviews were conducted at the NIH Clinical Center, and the remainder were performed by telephone. Subject characteristics are reported in Table 1. All subjects met criteria for clinical diagnosis of FD/MAS based on the presence of 2 or more characteristic features (Table 1). In addition, *GNAS* mutation testing from FD tissue was available for 15 subjects; in 7 subjects a mutation was identified at the R201C position, and in 8 subjects a mutation was identified at the R201H position. The mean total length of follow-up was 11.5 years (standard deviation 5.1 years, range 0–16 years).

**Table 1:** Subject characteristics

Number of subjects	39
Age at evaluation, median (range)	38 years (15–98)
Age at menarche, median (range)	3 years (0.5–13)
Fibrous dysplasia	97% (38)
McCune-Albright syndrome features, % (n)	
- Precocious puberty	90% (35)
- Café-au-lait macules	67% (26)
- Hyperthyroidism	38% (15)
- Growth hormone excess	26% (10)
- Hypophosphatemia	44% (17)
- Cushing syndrome	0

Pelvic ultrasounds were reviewed by a single reader (DOC). Biochemistries were drawn within 48 h of pelvic ultrasound. Biochemical assays were carried out by the Department of Laboratory Medicine at the NIH Clinical Center. From 2008 to 2015, LH and FSH were measured using Immulite (Diagnostic Products Corporation, Los Angeles, CA) competitive immunoassay. For LH, intra-assay coefficient of variation was 3.7%, inter-assay coefficient of variation was 6.7%, and lower limit of detection was 0.1 mIU/mL. For FSH, intra-assay coefficient of variation was 3.2%, inter-assay coefficient of variation was 4.8%, and lower limit of detection was 0.1 mIU/mL. Estradiol was measured using Roche Cobas e601 analyzer (Roche Diagnostics, Indianapolis, IN) electrochemiluminescence immunoassay. Intra-assay coefficient of variation was 1.9%, inter-assay coefficient of variation was 3.1%, and lower limit of detection was 4 pg/mL. For assays run prior to 2008, LH and FSH were measured using a Microparticle Enzyme Immunoassay (Abbott Laboratories, Abbott Park, IL). For FSH, intra-assay coefficient of variation was 4%, inter-assay coefficient of variation was 3%, and lower limit of detection was 0.37 mIU/mL. For LH, intra-assay coefficient of variation was 5%, inter-assay coefficient of variation was 4%, and lower limit of detection was 0.5 mIU/mL. Estradiol was measured using Immulite (Diagnostic Products Corporation, Los Angeles, CA) competitive immunoassay. Intra-assay coefficient of variation was 6.6%, and lower limit of detection was 15 pg/mL. In pre-menopausal women, levels vary widely according to menstrual cycle, but are typically in the range of 15–350 pg/mL for estradiol, 1–24 U/L for FSH, and 1–100 U/L for LH.

Statistical tests were performed using GraphPad Prism 7.03 (San Diego, CA). Comparisons between groups were performed using t-tests and Mann-Whitney tests, depending on the normality of the distribution. Results are reported using mean (standard deviation) or median (inter-quartile range)(IQR) as described, depending on the normality of the distribution.

## Results

### Abnormal uterine bleeding

Chronic abnormal uterine bleeding was defined as vaginal bleeding that is abnormal in regularity, volume, frequency, or duration, and has been present for the majority of at least 6 months [12, 13]. Most respondents (77%, 30/39) had a menstrual history consistent with chronic abnormal uterine bleeding. Of these, all 30 women reported bleeding that was abnormally high in frequency and volume, and 9 additionally reported bleeding of abnormally long duration. Thirteen women (33%) reported a history of anemia related to abnormal uterine bleeding. Nine of these 13 women received oral iron supplements for treatment of anemia, and 3 required ≥1 blood transfusion.

The most common treatment for abnormal uterine bleeding was oral contraceptive pills (83%, 25/30 subjects). This was reported to be effective in most subjects (72%, 18/25 treated subjects). Other treatments included levonorgestral intrauterine devices (*n* = 2, both effective), and combination estrogen/progestin patch (*n* = 1, ineffective).

Nine women underwent hysterectomy, all of whom had a history of abnormal uterine bleeding. The indications for hysterectomy were abnormal uterine bleeding alone in 6 women, abnormal uterine bleeding in the context of endometriosis in 2 women, and uterine prolapse in one woman. The age range at the time of hysterectomy was 27 to 44 years. Of note, 6/9 hysterectomies (67%) were performed in women at the unusually young age of < 35 years.

### Fertility

Fertility was assessed in the subset of women who had either achieved pregnancy, or reported ≥12 months of unprotected intercourse between ages 15 and 44. Out of 21 women in whom fertility status could be evaluated, 9 (43%) met criteria for infertility. This included 7 women who failed to achieve spontaneous pregnancy after 12 months of unprotected intercourse, and 2 women who carried a medical diagnosis of primary ovarian insufficiency. 8/9 women who met criteria for infertility (89%) had a history of MAS-associated precocious puberty.

One woman was diagnosed with primary ovarian insufficiency after presenting with secondary amenorrhea at age 16. Lab work at that time showed FSH 60 U/L, LH 40 U/L, and undetectable estradiol. She had a history of precocious puberty presenting at age 7 months, and underwent bilateral cystectomy at age 23 months. She continued to have symptoms of precocious puberty, and was treated with testolactone from ages 3 to 8. After discontinuing testolactone she had irregular menses until her presentation with secondary amenorrhea at age 16.

Another woman also presented with secondary amenorrhea at age 16 years, with a biochemical evaluation that showed FSH 61 U/L, LH 29 U/L, and undetectable estradiol. She had a history of precocious puberty presenting at age 6 months, at which time she developed by a large cyst complicated by ovarian torsion. She underwent unilateral salpingo-oophorectomy, and following surgery had no additional symptoms of precocious puberty, began spontaneous breast development starting at age 10, and regular menses starting at age 11. Neither woman reported environmental exposures or medications associated with ovarian toxicity. No autoimmunity or additional disorders associated with ovarian insufficiency were identified in either subject.

### Obstetrical outcomes

A total of 25 pregnancies occurred in 14 women. All pregnancies were spontaneous, without the use of assisted reproductive technology, and 8 (35%) were unplanned. Four pregnancies (18%) resulted in spontaneous abortion, and 6 (27%) resulted in therapeutic abortion. One pregnancy (4%) resulted in fetal demise due to placental abruption at 27 weeks gestation. A total of 14 live births occurred from 10 women. Thirteen of these births were full-term, and 1 was pre-term at 36 weeks gestation. Eight live infants were delivered vaginally (54%), and 6 were delivered by Caesarian section (46%). The indication for Caesarian section was described as fibrous dysplasia-related in 4 deliveries, and fetal status-related in 2 deliveries.

### Skeletal complications during menstruation and pregnancy

Menses were associated with no change in bone pain in 22 subjects (56%), increased bone pain in 12 subjects (31%), decreased bone pain in 0 subjects, with 5 subjects unsure. Among the 14 pregnancies, pregnancy was associated with no change in bone pain in 7 subjects (53%), increased bone pain in 4 subjects (31%), decreased bone pain in 2 subjects (15%), with 1 subject unsure. The postpartum period was associated with no change in bone pain in 10 subjects (71%), increased bone pain in 1 subject (7%), and decreased bone pain in 3 subjects (21%).

No additional skeletal complications, including aneurysmal bone cysts or malignant transformation, were reported during pregnancy.

### Radiographic and biochemical data

**Fig. 1:**
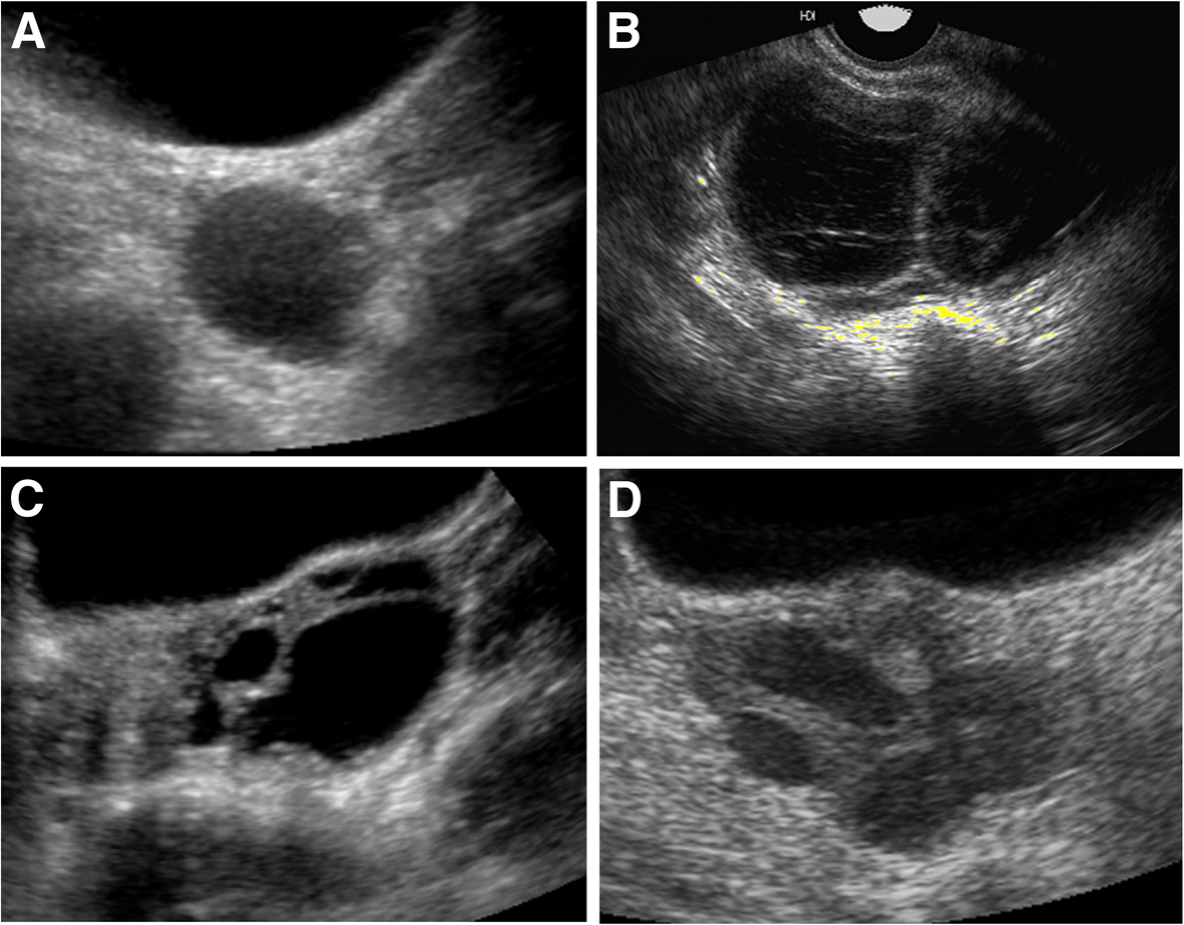
Representative pelvic ultrasound images. **a** Solitary cyst, measuring 4 cm in diameter. **b** Septated ovarian cyst, measuring 4 cm in diameter. **c** Multicystic adnexal mass, measuring 6 cm in diameter. **d** Multiple cysts involving the right ovary, the largest measuring 3 cm in diameter

Of the 39 total subjects, 19 had premenopausal radiographic and biochemical data available for review. Results of simultaneously obtained gonadotropins, estradiol levels, and pelvic ultrasonography are shown in Table 2 for the 14 of these subjects who were not receiving hormonal contraceptives at the time of the studies. The presence of follicles/cysts ≥2.5 cm in diameter are noted. Representative ultrasound images are shown in Fig. 1. Six subjects had multiple data points (corresponding to ≥1 admission to the NIH Clinical Center), and 8 subjects had a single data point. Gonadotropins were suppressed to undetectable levels in 6 women at 9 total timepoints; 8 of these timepoints showed evidence of ovarian cysts/follicles on ultrasound. The median estradiol levels for subjects with and without ovarian cysts was 139 pg/mL (IQR 68, 193) and 74 pg/mL (IQR 26, 140), respectively (*p* = 0.11). The median LH levels for subjects with and without ovarian cysts was 1.1 U/L (IQR 0.9, 3.3) and 2.6 U/L (IQR 0.8, 5.6), respectively (*p* = 0.17). The corresponding median FSH levels were 1.1 U/L (IQR 0.9, 5.2) and 2.5 U/L (IQR 0.6, 6.6), respectively (*p* = 0.46).

**Table 2:** Biochemical and ultrasonography findings from pre-menopausal women with McCune-Albright syndrome

Admission 1						Admission 2						Admission 3						Admission 4					
Age (y)	CD	LH (U/L)	FSH (U/L)	E2 (pg/mL)	Pelvic US	Age (y)	CD	LH (U/L)	FSH (U/L)	E2 (pg/mL)	Pelvic US	Age (y)	CD	LH (U/L)	FSH (U/L)	E2 (pg/mL)	Pelvic US	Age (y)	CD	LH (U/L)	FSH (U/L)	E2 (pg/mL)	Pelvic US
17	7	3.8	4.9	150	L, 9.5 cm	18	21	3.6	4.9	150	nl	20	22	6.4	7.8	64.7	nl	22	31	4.4	6.1	48.2	L, 4.8 cm
21	5	1	< 1	202	B, 5 cm	22	9	7	9	313	B, 1.9–4.4 cm	23	30	1	1	107	nl						
26	22	< 1	< 1	86	B, 3.6–4 cm	28	24	< 1	< 1	21	L, 5.8 cm	41	11	7.3	13	130	nl						
32	31	< 1	5	191	R, 2.5 cm	33	4	< 1	< 1	105	R, 3.5 cm	46	40	7.6	11.6	9.6	nl						
15	36	5.5	2.5	74	nl	16	31	0.7	0.2	447	nl	17	15	2.4	1.4	48.6	nl						
16	11	< 1	< 1	144	B, 3–6 cm	23	27	< 1	< 1	250	B, 4.3–5 cm												
18	13	< 1	< 1	74	R, 7.5 cm																		
22	2	2.6	3.3	224	nl																		
21	25	3	2	134	B, 2.5 cm																		
21	22	0.2	< 0.1	39.5	nl																		
26	14	1.6	0.2	< 5	nl																		
35	14	5.2	7.2	87	R, 3.8 cm																		
36	6	4.9	8.3	75	nl																		
31	19	2.7	4.7	179	R, 2.8 cm																		

*y* Years, *CD* Cycle day, *LH* Luteinizing hormone, *U/L* Units per liter, *FSH* Follicle stimulating hormone, *E2* Estradiol, *pg/mL* Picograms per milliliter, *US* Ultrasound, *B* Bilateral ovarian cysts, *L* Left-sided ovarian cyst, *R* Right-sided ovarian cyst, *cm* Centimeters, *nl* Normal

## Discussion

Findings from this large series demonstrate that autonomous ovarian activation in MAS persists into adulthood, with significant effects on gynecologic function and reproduction. In our cohort, abnormal uterine bleeding resulted in significant morbidity, including the need for blood transfusions in multiple subjects. This is consistent with previous case reports and small series reporting menometrorrhagia [14–17]. Treatment for abnormal uterine bleeding in this cohort included primarily oral contraceptive pills and surgical management. A striking finding was the unusually young age at which hysterectomies were performed. Most subjects were under age 35 years at the time of surgery, likely reflecting the severity of abnormal uterine bleeding. Subjects reported only infrequent use of levonorgestrel intrauterine devices, now considered first-line management for abnormal uterine bleeding in women who do not desire pregnancy [18]. It is possible that increased use of medical therapies that act locally at the level of the uterine endometrium, such as intrauterine devices or ulipristal acetate for women co-morbid leiomyomas, may improve gynecologic morbidity in women with MAS, potentially decreasing or preventing the need for surgical intervention [19].

Ovarian cysts persisted into adulthood in this cohort, resulting in hyperestrogenism and suppression of pituitary gonadotropin production in some patients. Implications of hyperestrogenism in women with MAS are unclear. MAS has recently been associated with an increased risk of breast cancer, particularly in younger women [20]. Precocious puberty has been identified as a risk factor for breast cancer within the MAS population, potentially reflecting the effects of long-term exposure to increased circulating estrogen.

This is the largest series to report data on fertility in women with MAS. Previous reports have been limited to single cases and small series, which have reported both spontaneous pregnancies [6, 8–10, 21–24] and infertility [16, 17, 25, 26]. Findings from this cohort included an infertility prevalence of 43%, which is substantially increased over the U.S. national average of 10.9% [27]. Thirty-five percent of pregnancies in this cohort were unplanned, which is only mildly decreased in comparison to the 49% U.S. national average [28]. Taken together, these findings suggest that fertility is impaired in women with MAS, however the possibility remains for spontaneous conception. The mechanism of infertility is likely related in part to anovulatory cycles resulting from autonomous ovarian activity [29], and it is possible that conception may not be impaired during intervals without ovarian activation. This could potentially account for the relatively robust prevalence of unplanned pregnancies in this cohort, highlighting the importance of contraceptive care in the MAS population. It should be noted that none of the women in this study sought reproductive assistance. Subjects were not specifically questioned about their decision-making, however concerns about skeletal effects of pregnancy and reproductive interventions may have played a role. It is likely that reproductive assistance may have increased pregnancy rates in this cohort, and should be recommended at an appropriate point to women with MAS who desire pregnancy.

If anovulation is the primary cause of infertility in MAS, ovarian cyst frequency may predict an individual’s degree of fertility impairment. There are 4 reports of women with frequent ovarian activation who underwent unilateral oophorectomy in attempts to improve contralateral ovarian function and fertility [16, 17, 25, 26]. All women reported improvement in menses, and 2 achieved spontaneous pregnancy. It is important to note that success with this approach may be predicated on the laterality of ovarian involvement, and that a unilateral oophorectomy may be less likely to improve contralateral function in women with bilateral disease. In addition, decreased ovarian reserve is an established risk of pelvic surgery [30], which is particularly relevant given the frequent and often inappropriate pelvic surgeries performed in women with MAS [31]. Two women in our series developed primary ovarian insufficiency after ovarian surgery, including one who had resolution of precocious puberty after oophorectomy, suggesting she had unilateral involvement. These findings suggest that unilateral oophorectomy may be helpful for treatment of MAS-associated infertility in select cases, however this approach should be undertaken with extreme caution with consideration of the potential for harm to fertility, and only under the supervision of an experienced reproductive specialist.

The effects of menstruation and pregnancy on FD has been an area of much speculation and concern for patients. Case reports have described aneurysmal bone cysts and malignant transformation in pregnant women, fueling conjecture that pregnancy hormones may affect adversely affect the metabolic activity of FD lesions [6–11]. Osada et al. reported increased bone turnover during pregnancy in women with FD, however these changes were consistent with the bone turnover variations typical of pregnancy and the post-partum period [21]. Skeletal complications are an established morbidity in FD, and it is possible that their reported association with pregnancy is mediated in part by reporting bias, due to the current literature dominated by case reports. In this cohort study, there were no consistent effects of pregnancy or menstruation on bone pain, and no association with skeletal morbidity. Currently there is no clear evidence to establish a causative relationship between pregnancy or menstruation and skeletal outcomes in women with FD/MAS.

Strengths of this study include the large number of subjects and extensive follow-up. This is the first cohort study to establish gynecologic disease as a highly prevalent source of morbidity in MAS, to evaluate fertility and reproductive outcomes in women with MAS, and to report on skeletal outcomes in pregnancy. This is also the first report of primary ovarian insufficiency as a potential consequence of pelvic surgery in MAS, a novel finding that may directly inform gynecologic management. Limitations include the retrospective nature of the study design. Clinical outcomes data were not collected at same time as radiographic data and biochemical data, and are subjected to recall bias. Interpretation of ultrasound and biochemical data is limited, because studies were obtained in a cross-sectional manner and were not standardized according to menstrual cycles. Additional, prospective studies are needed to characterize the prevalence and frequency of ovarian cysts, which will provide important context for these clinical findings. Prospective studies should correlate gynecologic data with skeletal outcomes and surrogate endpoints, such as bone turnover markers, to more fully investigate the effects of these hormonal changes on mineral metabolism. Selection bias likely resulted in over-representation of more severely affected patients among respondents. The high prevalence of precocious puberty among respondents likely reflects this selection bias. This data should therefore be generalized only to patients with known MAS ovarian involvement.

## Conclusions

Abnormal uterine bleeding is common in MAS and may result in significant morbidity, including severe anemia and hysterectomies at an early age. In this series, the prevalence of infertility was substantially increased over the national average, however the prevalence of unplanned pregnancies was only mildly reduced. Menses and pregnancy were not consistently associated with increased FD-related bone pain or skeletal complications. These findings will inform family planning for patients with FD/MAS by highlighting both the potential for spontaneous pregnancy, as well as possibility of impaired fertility and need for reproductive assistance.

## Abbreviations

FD: Fibrous dysplasia
FSH: Follicle stimulating hormone
LH: Luteinizing hormone
MAS: McCune-Albright syndrome
NIDCR: National Institute of Dental and Craniofacial Research
NIH: National Institutes of Health

## References

[1] Weinstein LS (1991). Activating mutations of the stimulatory G protein in the McCune-Albright syndrome. N Engl J Med.

[2] Boyce AM, Adam MP (1993). Fibrous dysplasia/McCune-Albright syndrome. GeneReviews((R)).

[3] Collins MT, Singer FR, Eugster E (2012). McCune-Albright syndrome and the extraskeletal manifestations of fibrous dysplasia. Orphanet J Rare Dis.

[4] Schoelwer M, Eugster EA (2016). Treatment of peripheral precocious puberty. Endocr Dev.

[5] Estrada A (2016). Long-term outcomes of letrozole treatment for precocious puberty in girls with McCune-Albright syndrome. Eur J Endocrinol.

[6] Kaplan FS (1988). Estrogen receptors in bone in a patient with polyostotic fibrous dysplasia (McCune-Albright syndrome). N Engl J Med.

[7] Stevens-Simon C (1991). Exacerbation of fibrous dysplasia associated with an adolescent pregnancy. J Adolesc Health.

[8] Mintz MC, Dalinka MK, Schmidt R (1987). Aneurysmal bone cyst arising in fibrous dysplasia during pregnancy. Radiology.

[9] Bowers CA (2012). Pregnancy-induced cystic degeneration of fibrous dysplasia. Can J Neurol Sci.

[10] Kanazawa I (2009). Osteosarcoma in a pregnant patient with McCune-Albright syndrome. Bone.

[11] Bhattacharya S, Mishra RK (2015). Fibrous dysplasia and cherubism. Indian J Plast Surg.

[12] Munro MG (2011). FIGO classification system (PALM-COEIN) for causes of abnormal uterine bleeding in nongravid women of reproductive age. Int J Gynaecol Obstet.

[13] American College of Obstetricians and Gyne-cologists. Practice bulletin no. 128: diagnosis of abnormal uterine bleeding in reproductive-aged women. Obstet Gynecol. 2012;120(1):197–206. 10.1097/AOG.0b013e318262e320 22914421

[14] Lala R (2007). Persistent hyperestrogenism after precocious puberty in young females with McCune-Albright syndrome. Pediatr Endocrinol Rev.

[15] Matarazzo P (2006). McCune-Albright syndrome: persistence of autonomous ovarian hyperfunction during adolescence and early adult age. J Pediatr Endocrinol Metab.

[16] Laven JS (2004). Management of infertility in a patient presenting with ovarian dysfunction and McCune-Albright syndrome. J Clin Endocrinol Metab.

[17] Chanson P, Salenave S, Young J (2010). Ovarian dysfunction by activating mutation of GS alpha: McCune-Albright syndrome as a model. Ann Endocrinol (Paris).

[18] American College of Obstetricians and Gyne-cologists. Practice bulletin no. 136: management of abnormal uterine bleeding associated with ovulatory dysfunction. Obstet Gynecol. 2013;122(1):176–85. 10.1097/01.AOG.0000431815.52679.bb 23787936

[19] Donnez J (2012). Ulipristal acetate versus leuprolide acetate for uterine fibroids. N Engl J Med.

[20] Majoor Bas CJ, Boyce Alison M, Bovée Judith VMG, Smit Vincent THBM, Collins Michael T, Cleton-Jansen Anne-Marie, Dekkers Olaf M, Hamdy Neveen AT, Dijkstra PD Sander, Appelman-Dijkstra Natasha M (2017). Increased Risk of Breast Cancer at a Young Age in Women with Fibrous Dysplasia. Journal of Bone and Mineral Research.

[21] Osada H (2005). Accelerated bone turnover in pregnant women with McCune-Albright syndrome. Gynecol Obstet Investig.

[22] Wong SC, Zacharin M (2017). Long-term health outcomes of adults with McCune-Albright syndrome. Clin Endocrinol.

[23] Lee PA, Van Dop C, Migeon CJ (1986). McCune-Albright syndrome. Long-term follow-up. JAMA.

[24] Malchoff CD (1994). An unusual presentation of McCune-Albright syndrome confirmed by an activating mutation of the Gs alpha-subunit from a bone lesion. J Clin Endocrinol Metab.

[25] Chevalier N (2015). Postpubertal persistent Hyperestrogenemia in McCune-Albright syndrome: unilateral oophorectomy improved fertility but detected an unexpected borderline epithelial ovarian tumor. J Pediatr Adolesc Gynecol.

[26] Lavoue V (2008). Restoration of ovulation after unilateral ovariectomy in a woman with McCune-Albright syndrome: a case report. Eur J Endocrinol.

[27] Chandra A, Copen CE, Stephen EH. Infertility and impaired fecundity in the United States, 1982-2010: data from the National Survey of family growth. Natl Health Stat Report. 2013;(67):1–18, 1 p following 19. 24988820

[28] Finer LB, Zolna MR (2011). Unintended pregnancy in the United States: incidence and disparities, 2006. Contraception.

[29] Laven JS (2001). Dynamics of ovarian function in an adult woman with McCune--Albright syndrome. J Clin Endocrinol Metab.

[30] Alammari R, Lightfoot M, Hur HC (2017). Impact of cystectomy on ovarian reserve: review of the literature. J Minim Invasive Gynecol.

[31] Nabhan ZM, West KW, Eugster EA (2007). Oophorectomy in McCune-Albright syndrome: a case of mistaken identity. J Pediatr Surg.

